# Decreased therapeutic effects of noscapine combined with imatinib mesylate on human glioblastoma in vitro and the effect of midkine

**DOI:** 10.1186/1475-2867-11-18

**Published:** 2011-06-08

**Authors:** Mine Erguven, Ayhan Bilir, Nuray Yazihan, Ezgi Ermis, Akin Sabanci, Esin Aktas, Yavuz Aras, Vehbi Alpman

**Affiliations:** 1 Yeni Yüzyıl University, Faculty of Medicine, Department of Biochemistry, Istanbul, Turkey; 2 İstanbul University, Istanbul Faculty of Medicine, Department of Histology and Embryology, Istanbul, Turkey; 3 Ankara University, Faculty of Medicine, Department of Pathophysiology, Ankara, Turkey; 4 Ankara University, Faculty of Medicine, Institute of Molecular Biology and Genetics, Ankara, Turkey; 5 Girne Asker Hastanesi, Kyrenia Military Hospital, Department of Neurosurgery, Girne, Cyprus; 6 Istanbul University, Institute of Experimental Medicine (DETAE), Department of Immunology, Turkey; 7 Istanbul University, Istanbul Faculty of Medicine, Department of Neurosurgery, Istanbul, Turkey; 8 Yeni Yüzyıl University, Faculty of Medicine, Department of Neurology, Istanbul, Turkey

## Abstract

**Background:**

Glioblastoma (GBM) develops resistance to the advances in chemotherapy leading to poor prognosis and life quality. Consequently, new treatment modalities are needed. Our aims were to investigate the effects of combined noscapine (NOS) and imatinib mesylate (IM) on human GBM *in vitro *and the role of midkine (MK) in this new combination treatment.

**Methods:**

Monolayer and spheroid cultures of T98G human GBM cell line were used to evaluate the effects of IM (10 μM), Nos (10 μM) and their combination on cell proliferation and apoptotic indexes, cell cycle, the levels of antiapoptotic MK, MRP-1, p170, PFGFR-α, EGFR, bcl-2 proteins, apoptotic caspase-3 levels, morphology (SEM) and ultrastructure (TEM) for 72 hrs. Results were statistically analyzed using the Student's t-test.

**Results:**

The combination group induced highest decrease in cell proliferation and apoptotic indexes, caspase-3 levels, MRP-1 and PDGFR-α levels. The decrease in p170 levels were lower than IM but higher that NOS. The highest increases were in EGFR, MK, bcl-2 and cAMP levels in the combination group. The G0+G1 cell cycle arrest at the end of 72^nd ^hr was the lowest in the combination group. Apoptotic appearence was observed rarely both in the morphologic and ultrastructural evaluation of the combination group. In addition, autophagic vacuoles which were frequently observed in the IM group were observed rarely.

**Conclusions:**

The combination of Nos with IM showed antagonist effect in T98G human GBM cells in vitro. This antagonist effect was correlated highly with MK levels. The effects of NOS on MRP-1, MK and receptor tyrosine kinase levels were firstly demonstrated in our report. In addition, we proposed that MK is one of the modulator in the switch of autophagy to cell death or survival/resistance.

## Background

Glioblastoma (GBM) is the most common and malignant primary brain tumor. Although various combined therapies of surgery, radiation and chemotherapy are tried in order to cope with resistance and relapse to prolong survival time, the prognosis of patients remains poor. The majority of the patients die within a year of diagnosis and the five-year-survival of patients worldwide with glioma is only 10% [1].

In the chemotherapy era, this shortcoming leads investigators to design new antineoplastic agents and to use them alone or in combination with previously-used agents. Although these advances achieved some success, this process from bench to in vivo and phase trials took considerable time and financial resources, resulting in more deaths. Consequently, investigators started to search whether or not, commonly and effectively used non-antineoplastic drugs long-used in clinic had antineoplastic effects and/or ability to potentiate antineoplastic drug cytotoxicity [2,3]. A plant alkaloid with well-known antitussive effects Noscapine (Nos) was investigated in these trials and Nos was proposed as a promising antineoplastic agent. The anticancer action of Nos involves the induction of cell cycle arrest via attenuation of microtubule dynamics through activation of mitotic checkpoints. It has been shown that Nos binds stoichiometrically to tubulin and then promotes microtubule polymerization but does not alter the steady state monomer/polymer ratio of tubulin, and consequently causes growth arrest of tumor cells during mitosis, and also reverses P-gp mediated drug resistance [4,5]. In addition, researchers chose to investigate current antineoplastic agents when their success was proven for specific cancer types or for other cancer types. These antineoplastic agents target the same pathways but are often affected negatively by different mutations such as IM in chronic myeloid leukemia and GBM treatments [6,7].

IM is a tyrosine kinase inhibitor as a platelet-derived growth factor receptor (PDGFR) interacting with the adenosine triphosphate (ATP)-binding site. IM was initially designed to treat chronic myelogenous leukemia but the success of this compound in this treatment led to broader application in the treatment of other tumors, such as gastrointestinal tumours, anaplastic thyroid cancer, prostate cancer and gliomas [6-10]. Previous reports demonstrated that monotherapy by using IM is efficacious in in vitro models of GBM because of its potency to inhibit the signaling pathway of PDGFR [11]. Although it was shown that monotherapy with IM is also effective in *in vitro *models of GBM, IM could not achieve the same success as in *in vivo *models because of its poor brain distribution [12]. Unfortunately, in accordance with *in vivo *studies, monotherapy with IM has minimal activity in Phase II studies of GBM. IM did not show the dramatic results that are sometimes seen with other targeted therapies such as IM in CML [13]. The reason explained by previous studies was that IM is a substrate of two efflux pumps P-glycoprotein (P-gp) and breast cancer resistance protein (BCRP1) which are excreted by the drug from the cell and expressed at the blood-brain barrier (BBB) [13,14].

Midkine (MK), a heparin-binding growth factor, was originally reported to be the product of a retinoic acid-responsive gene during embryogenesis [15]. Its expression is high during embryogenesis, but interestingly, MK is undetectable in healthy adults and only reappears in the body as a part of disease pathogenesis. Moreover, the most intriguing feature of MK is its high-frequency and massive expression in advanced tumors [16-19]. Previous reports showed that increased levels of MK expression correlate with the progression of human astrocytomas, MK mRNA and protein expression levels were higher in high-grade astrocytomas (anaplastic astrocytomas and GBMs) than in low-grade astrocytomas [20].

Previous reports showed that Nos led to a potent inhibition of cell proliferation and induced apoptosis in glioma/GBM cell lines in vitro and in vivo [21,22]. In addition it was shown that Nos reversed P-gp induced resistance through c-jun-NH2-terminal kinase (JNK) in human gastric and pancreatic carcinomas [5]. In the present study, we investigated primarily whether Nos could potentiate the cyctotoxicity of IM in human GBM cells in vitro and secondly we also investigated the role of MK as a survival and resistance molecule in this combination treatment. We thought MK can involve in the fate of this new combination chemotherapy.

## Methods

### Monolayer and spheroid cell cultures

T98G human GBM cell line was provided by the American Type Culture Collection (ATCC; Rockville, USA) and was grown in monolayer culture in Dulbecco's Modified Eagle's Medium-F12 (DMEM-F12; Biological Industries, Israel) supplemented with 10% heat-inactivated foetal calf serum, 1 mM sodium pyruvate, 0.1 mM non-essential aminoacid solution, 50 units/ml penicillin and streptomycin (Sigma Chemical Co., St Louis, Missouri). Cells in semiconfluent flasks were harvested using 0.05% trypsin, 0.53 mM EDTA solution (Sigma Chemical Co., St Louis, Missouri) and centrifuged after the addition of DMEM-F12 for trypsin inactivation and then resuspended in culture medium. Following trypan blue exclusion assay, GBM cells were plated in six-well culture plates containing 5 ml DMEM-F12 medium at a concentration of 5 × 10^5 ^cells/well with 100% vitality.

An in vitro multicellular T98G GBM spheroid model was established using a liquidoverlay technique. Briefly, semi-confluent monolayer cell cultures were trypsinized and single cells with 100% vitality were cultured over 3% Noble agar-coated (Difco, USA) six-well culture plates containing 5 ml DMEM-F12medium at a concentration of 1 × 10^6 ^cells/well.

### Experimental design

IM and Nos were applied at a volume of 100 μl to monolayer cultures of T98G GBM cells in concentrations ranging from 1 μM to 200 μM, while the negative control cells received only nutrient medium alone. Cultures were incubated with drugs for 72 hrs. The inhibition concentration 50 (IC_50_) values were determined as 10 μM for two drugs. Experiments were performed in monolayer and spheroid cultures of T98G human GBM cells. Groups were identified as control, IM (10 μM), Nos (10 μM) and their combination. For every experimental group n = 6. All experiments were repeated three times and achieved similar results. Cell proliferation index (total cell number by propidium iodide (PI) staining), apoptotic index by flow cytometric (FCM) Annexin-V-fluorescein isothiocyanate/PI (Annexin-V-FITC/PI) staining, cell cycle by (FCM), the levels of caspase-3, MK, epidermal growth factor receptor (EGFR) by enzyme-linked immunosorbent assay (ELISA), the levels of bcl-2, multi-dug resistance protein-1 (MRP-1), p170, PDGFR alpha (PDGFR-α) by Western blotting, cAMP levels by radioimmunoassay (RIA), morphology by scanning electron microscopy (SEM) were evaluated in monolayer cultures for 72 hrs. In addition, cell ultrastructure was evaluated in spheroid cultures by using transmission electron microscopy (TEM) for 24 hrs.

### Cell proliferation index

The total cell number was counted by using an automated cell counter (nucleocounter, Denmark). The starter kit which is compatible to cell counter and includes lysis buffer, stabilization buffer, nucleocasettes and software was used. Cells were harvested every 24 hrs for 72 hrs. Cells were pre-treated with lysis and stabilization buffers to dissolve cell aggregates and lyse cell membranes. Pre-treated cells were loaded to nucleocasettes which were coated with propidium iodide (PI) dye and their nuclei were stained with PI. Nucleocasettes were placed in device for 30-35 seconds to measure the PI fluorescence and then cell counts were analyzed with the software and recorded.

### Apoptotic index

One of the manifestations of apoptosis is the translocation of phosphotidylserine (PS) from the inner membrane to the outer side of the plasma membrane. Externalization of PS was studied by the Annexin-V-binding assay. Briefly, cells were washed twice with PBS and resuspended by binding buffer containing 0.01 M HEPES, 0.14 mM NaCl and 2.5 mM CaCl_2_. A cell suspension (1 × 10^5 ^cells in 100 μl) in binding buffer was incubated with 5 μl of FITC-labeled Annexin V (BD Pharmingen, San Diego, CA, USA) dye and propidium iodide (PI) for 15 mins in the dark at room temperature. After incubation, the PI fluorescence and Annexin V were measured simultaneously in a BD FACS Calibur and analyzed with the instrument's operating software (CellQuest: BD Pharmingen, San Diego, CA, USA). Data acquisition and analysis were undertaken with CellQuest and WinMDI programs.

### Caspase-3 levels

Cell culture supernatants were analyzed for caspase-3 levels in triplicate, using fluorimetric kits (Sigma Aldrich, Missouri, USA). The caspase-3 fluorimetric assay is based on the hydrolysis of the peptide substrate acetyl-Asp-Glu-Val-Asp-7-amido-4-methylcoumarin (Ac-DEVD-AMC) by caspase 3, resulting in the release of the fluorescent 7-amino-4-methylcoumarin (AMC) moiety. 1 × 10^4 ^cells seeded in each well of 96 well plates washed twice in PBS and incubated in CHAPS lysis buffer at 4°C for 20 mins. 5 μl cell lysate was transfered into the wells of other 96 well plates then incubated with 5 μl of 2 mM Ac-DEVD-pNA peptide substrate and 200 μl of assay buffer (HEPES 20 mM, pH 7.4, CHAPS 0.1%, DTT 5 mM, EDTA 2 mM) at 37°C for 1 hr in an incubator. The concentration of AMC released was quantified by reading in a fluorometer with a 360 nm excitation filter and 460 nm emission filter for optimal sensitivity.

### Cell cycle

The effects of drugs on the cell cycle were examined using a DNA analysis kit (BD Pharmingen, San Diego, CA, USA) according to the manufacturer's instructions. Briefly, T98G GBM cells were induced at a cell density of 5 × 10^5 ^cells/ml in the presence of each drug applied separately and in combination for different time intervals (24 and 72 hrs). Cells were then harvested, centrifuged, washed and resuspended in buffer (dimethylsulfoxide in sucrose-sodium citrate) for 5 mins at room temperature. A mixture of trypsin in spermine tetrahydrochloride detergent buffer was added and samples were incubated for 20 mins at room temperature. After the addition of RNase A and trypsin inhibitor in spermine buffer, cells were incubated with propidium iodide, in dark, for 20 mins at 4°C. Finally, flow cytometric analysis was performed immediately using a Facscan flow cytometer (FACS Diva, Beckman-Dickinson, California, USA) and fluorescence intensity data were acquired using the instrument's operating software (CellQuest: BD Pharmingen, San Diego, CA, USA). The percentages of the analyzed cell population in G0/G1-, S-, or G2/M-phases were determined by the Mod Fit cell-cycle analysis program.

### MK levels

Cell culture supernatants were analyzed for MK levels in triplicate, using ELISA kits (Peprotech). The lower detection limit of the assay was 150 pg/ml for MK. MK levels were measured by an ELISA system in which polyclonal antihuman MK was used as capture antibody (Peprotech). Detection was bybiotinylated polyclonal antihuman MK antibody (Peprotech) followed by streptavidin HRP (Sigma) and a TMB enzyme substrate system (Sigma). The reaction was stopped by 1 M H_2_SO_4 _and readings were made at 450 nm by a spectrometer (M2, Molecular devices, CA, USA).

### cAMP levels

Following centrifugation, the supernatant was removed and 0.1 N HCl with DMEM-F12 medium (1:1) was added to cells to stop the reaction at each 24 hrs interval. Briefly, 25 μl of the samples were used to measure cAMP levels. cAMP accumulation was measured in the supernatants according to the method previously described with some modifications [23]. cAMP was determined by RIA using the acetylation protocol. High-affinity rabbit anti-cAMP antibodies were raised in our laboratory using BSA-conjugated cAMP. Succinyl-cAMP tyrosine methylester (ScAMP-TME) was iodinated by the chloramine-T method. Mono- and diiodo ScAMP-TME were used as tracer ligands for the radioimmunoassay (RIA) and then purified by gel-filtration chromatography (Sephadex G25 superfine), equilibrated and eluted with 1 M sodium acetate (pH:5).

### EGFR levels

Cell culture supernatants were analyzed for EGFR levels in triplicate, using ELISA kits (SABiosciences, Germany). EGFR levels were measured by an ELISA system in which antihuman EGFR was used as capture antibody (SABiosciences, Germany) and detection was by biotinylated polyclonal antihuman EGFR antibody (SABiosciences) followed by streptavidin HRP (SABiosciences, Germany) and a TMB enzyme substrate system (SABiosciences, Germany). The reaction was stopped by 2 M H_2_SO_4 _and readings were made at 450 nm by a spectrometer (M2, Molecular devices, CA, USA).

### PDGFR-α, MRP-1, Bcl-2 and p170 levels by western blotting

Expression of PDGFR-α, MRP-1, Bcl-2, p170 proteins were detected by Western blot analysisas described in previous reports. 2 × 10^6 ^cells were lysed for 15 mins at 4°C in RIPA lysis buffer. Protein content was assessed using BCA protein assay (Pierce, Rockford, USA). Samples with equal amounts of protein were separated on a 7 and 10% sodium dodecyl sulfate polyacrylamide gelelectrophoresis (SDS-PAGE) gel, transferred to polyvinylidene fluoride (PVDF) membranes detected with PDGFR-α, MRP-1, bcl-2, p170 1/100 dilution and all from Santa Cruz Biotechnology, CA, USA and β-actin (1/1000 dilution, Pierce, Rockford, USA) antibodies. Protein bands were visualized with the enhanced chemiluminescence (ECL) Advance Western blot detection reagents (GE Healthcare Life Sciences, NJ, USA) and quantified by the ImageJ image processing program (National Institutes of Health, Bethesda, MD).

### Morphology by SEM

T98G GBM cells were seeded on microslides in 24-well culture plates containing 1 ml of DMEM-F12 medium at a concentration of 5 × 10^4 ^cells/well. After cell attachment, drugs at a concentration of 10 μM were applied to the cell cultures individually and in combination. Cells were fixed with 2.5% glutaraldehyde in 0.1 M of sodium cacodylate buffer (pH: 7.4) for 1 hr at 4°C. Cells were washed twice for 10 mins with 0.1 M of sodium cacodylate buffer and post-fixed in 1% osmium tetraoxide for 1 hr at 4°C. The cells were dehydrated in a graded acetone series and incubated in amyl acetate. Microslides were critical-point dried, sputter coated with gold-palladium and observed by SEM (Jeol-JSM-5200). Photographs were taken at several magnifications.

### Ultrastructure by TEM

Harvested spheroids were fixed with 2.5% glutaraldehyde in 0.1 M sodium cacodylate buffer and post-fixed in 1% osmium tetraoxide in 0.1 M sodium cacodylate buffer for 1 hr at 4°C. Cells were incubatedin 1% uranyl acetate for 1 hr at 4°C, dehydrated in a graded acetone series and embedded in Epon 812. Samples were cut using a rotating blade microtome (Leica, Heerbrugg, Switzerland) and 70 nm-thick sections were mounted on copper grids. Sections were subsequently stained with 5% uranyl acetate and counterstained with Reynold's lead citrate. Sections were examined with a Jeol-Jem 1011 TEM. Photographs were taken at several magnifications.

### Statistical analysis and determination of synergism

SPPS 17.0 statistical software (SPSS, Inc., Chicago, IL, USA) was used for the statistical analysis. All the results were statistically analyzed using the Student's t-test. Data were represented as mean ± standard error mean (SEM). p<0.05 was considered significant. Synergy was determined as described previously [24-26]. Briefly, synergism was determined using the following formula: Combination index (CI): D1/(DX)1+ D2/(DX)2, where D1 is tested concentration of IM used in combination with Nos, D2 is the tested concentration of Nos used in combination with IM, (DX)1 is the concentration of a singly applied IM and (DX)2 is the concentration of a singly applied Nos. A CI value of 1 indicates an additive effect, a CI value < 1 indicates a synergistic effect and a CI value > 1 indicates an antagonist effect.

## Results

### Cell proliferation index

**Figure 1:**
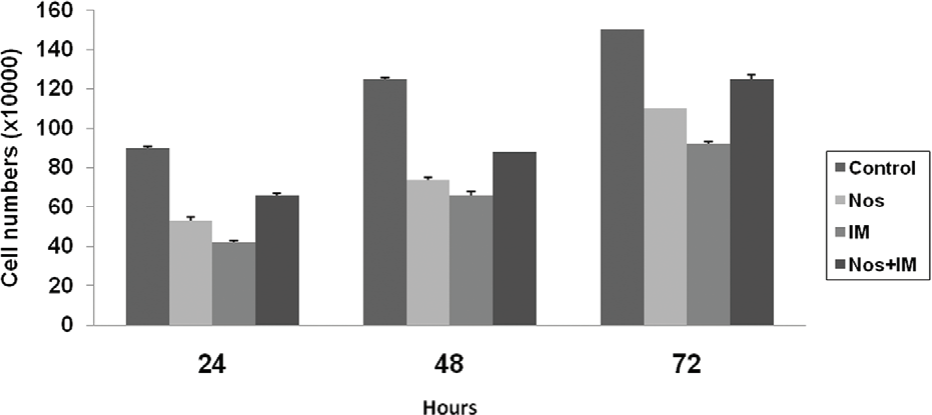
**Cell proliferation index**. Nos, noscapine; IM, imatinib mesylate; Nos+IM, noscapine with imatinib mesylate. Results are mean of three different experiments and are presented as mean ± standard error mean (SEM).

The effects of drug applications are summarized in Figure 1. The cell number of the control group showed a proportional increase as a function of incubation time up to 72 hrs. All drug treatments inhibit cell proliferation of T98G GBM cell lines for 72 hrs (p < 0.05) in a time-dependent manner. The highest decrease was determined at IM, Nos and the combination group, respectively (p < 0.05).

### Apoptotic index and caspase-3 levels

**Figure 2:**
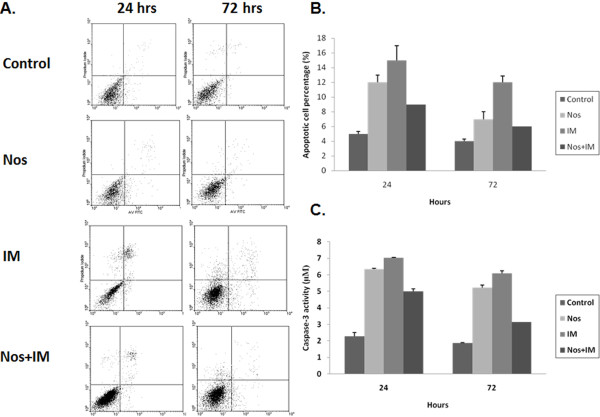
**Apoptotic index**. (**A)**The panels of flow cytometric analysis of apoptotic index. According to kits' instruction manual, quadrants of panels were defined as: the lower left quadrant of density plots for the numbers of viable cells (annexin V-; PI-), the lower right quadrant for the numbers of earlier stages of apoptotic cells (annexin V+; PI-), the upper right quadrant for the numbers of late stages of apoptotic cells (annexin V+; PI+), and the upper left quadrant for the numbers of dead cells. In the graph only the percentage of apoptotic cells (lower left and upper left) were presented.** (B) **The graph of apoptotic index. The columns in the graph represent the sum of earlier and late stages of apoptotic cells.**(C) **The graph of caspase-3 levels. Nos, noscapine; IM, imatinib mesylate; Nos+IM, noscapine with imatinib mesylate. Results are the mean of three different experiments and presented as mean ± SEM.

Figure 2 clearly shows different levels of apoptosis (Figure 2A) and caspase-3 activity (Figure 2B) induced by these drug applications. Nos, IM and the combination group increased apoptosis and caspase-3 activity at the 24^th ^hr and the 72^nd ^hr (p < 0.05). IM was the potent apoptosis inducer among other drug applications and the latter was Nos (p < 0.05).

### Cell cycle

**Figure 3:**
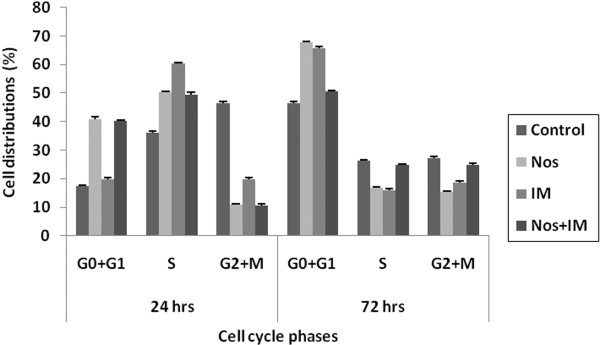
**Cell cycle**. The percentage of cells at G0/G1-, S- and G2/M-phases are plotted (%). Nos, noscapine; IM, imatinib mesylate; Nos+IM, noscapine with imatinib mesylate. Results are the mean of three different experiments and presented as mean ± SEM.

T98G GBM cells demonstrated growth arrest at the S-phase within 24 h of drug treatments but they demonstrated G0+G1 arrest at the 72^nd ^hr. IM was the most potent drug to induce S-phase arrest at the 24^th ^hr and Nos was apotent G0+G1 phase arrest inducer at the 72^nd ^hr (p < 0.05) (Figure 3).

### MK levels

**Figure 4:**
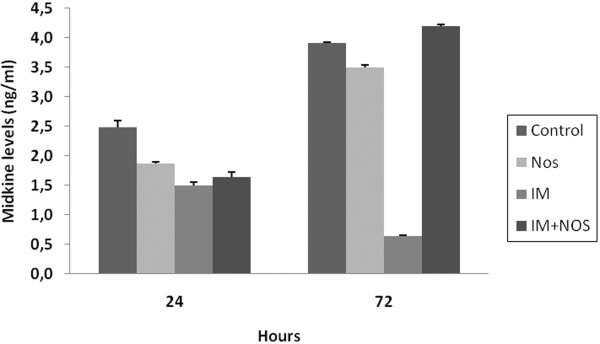
**Midkine levels**. Nos, noscapine; IM, imatinib mesylate; Nos+IM, noscapine with imatinib mesylate. Results are mean of three different experiments and presented as mean ± SEM.

The alterations in MK levels for 72 hrs are summarized in Figure 4. All drugs alone and incombination decreased MK levels at the 24^th ^hr, However, only IM and Nos decreased these levels at the 72^nd ^hr (p < 0.05). The combination group increased these levels at the 72^nd ^hr (p < 0.05).

### cAMP levels

**Figure 5:**
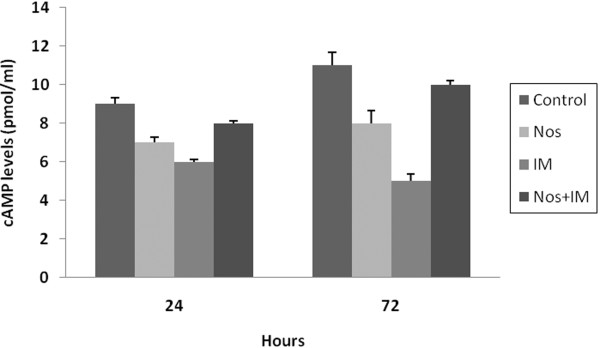
**cAMP levels**. Nos, noscapine; IM, imatinib mesylate; Nos+IM, noscapine with imatinib mesylate. Results are mean of three different experiments and presented as mean ± SEM.

cAMP levels were measured to assess the metabolic activity, growth and survival of cells. The cAMP level of the untreated control group culture increased from culture time zero to 72 hrs (p < 0.05)(Figure 5). The drug treatments reduced cAMP levels both individually and in combination (p < 0.05). The highest decrease was determined at IM, Nos and the combination groups, respectively (p < 0.05).

### EGFR levels

**Figure 6:**
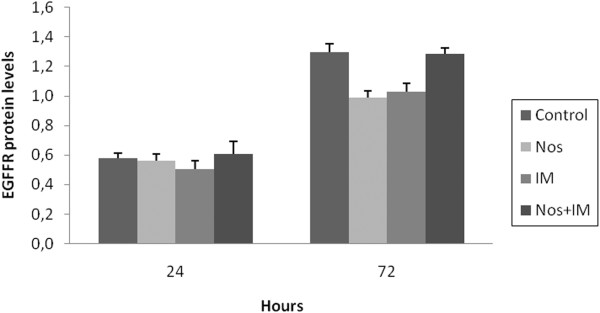
**EGFR levels**. Nos, noscapine; IM, imatinib mesylate; Nos+IM, noscapine with imatinib mesylate. Results are the mean of three different experiments and presented as mean ± SEM.

EGFR levels of control group were almost the same with Nos group (p > 0.05) at the 24^th ^hr. IM induced much higher inhibition than Nos(p < 0.05) and the combination group led to little increase at the 24^th ^hr (Figure 6). EGFR levels for Nos and IM were similar (p > 0.05) and they led to a sharp decrease at the 72^nd ^hr (p < 0.05). EGFR levels of the combination group were almost the same with the control group (p > 0.05).

### PDGFR-α, MRP-1, Bcl-2 and p170 levels by western blotting

**Figure 7:**
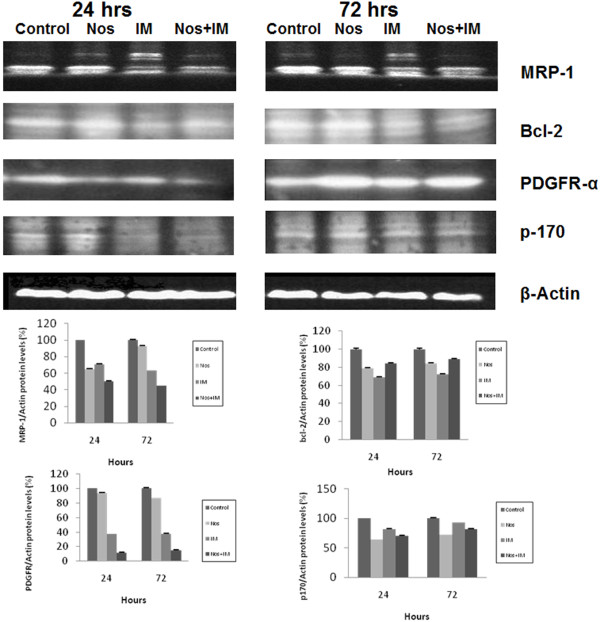
**PDGFR-α, MRP-1, Bcl-2 and p170 levels by western blotting**. Nos, noscapine; IM, imatinib mesylate; Nos+IM, noscapine with imatinib mesylate. Results are presented as mean (%) ± SEM. Mean values for each group were calculated as the ratio of the density of the sample band to that of the β-actin band in each lane and multipled with 100 and they are the mean of three different experiments.

All drugs alone and in combination decreased PDGFR levels, MRP-1 levels and bcl-2 levels (p < 0.05) (Figure 7). The combination group was very potent in decreasing PDGFR and MRP-1 (p < 0.05), the latter was IM (p < 0.05). Bcl-2 levels were decreased potently by IM, Nos and the combination group, respectively (p < 0.05).

### Morphology

**Figure 8:**
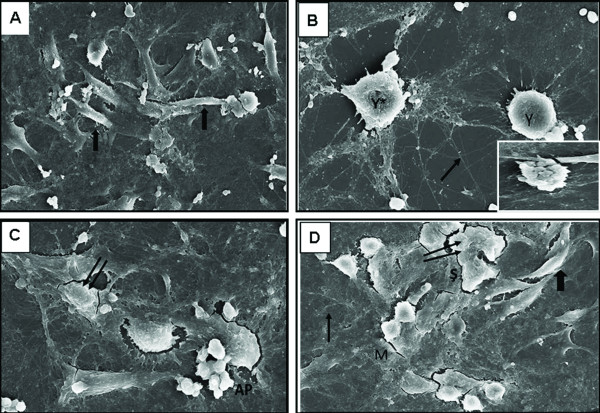
**Scanning electron microscopic views of T98 GBM cells at the 24^th ^hr**. **(A) **The control group (original magnification, ×1.5 k). Thick Arrow (**➜**) indicates healthy cell with thin and long body. Apoptotic appearence of T98G cell were shown in a small micrograph. (**B) **The IM group (original magnification of big and small electron micrographs are ×2.5 k and ×2.25 k, respectively). Thin arrow (→) indicates peaky and long microvillus of the cell. Y*, round cell which lost microvillus; Y: round cell with microvillus.(**C) **The Nos group. Two arrows represent cells which possess extended and angular body with protruding surface. AP, apoptotic appearence (original magnification, ×3.0 k). (**D) **The combination group (original magnification, ×1.80 k). Thick Arrow (**➜**) indicates healthy cell with thin and long body. Thin arrow (→) indicates peaky and long microvillus of the cell. Two arrows represent cells which possess extended and angular body with protruding surface. S, cell with extended body without a shape.

The control group of C6 glioma cells had a spindle-shaped appearance and frequent, active mitotic division phase was observed under SEM (Figure 8A). IM led to a sharp decrease in the number of microvilli and many cells changed to possess a globular appearance like a ball. Apoptotic appearence like grape grains was fequently observed (Figure 8B). In the Nos group many cells possessed a wide body with an angular morphology and a protruding surface because of cell surface knobs and apoptotic appearence was also observed (Figure 8C). In the combination group, two different cell types were observed as follows: i) a wide body with an angular morphology and a protruding surface, ii) a wide body with s spindle-shaped morphology. Mitotic cell appearence was also observed in this group (Figure 8D).

### Ultrastructure

**Figure 9:**
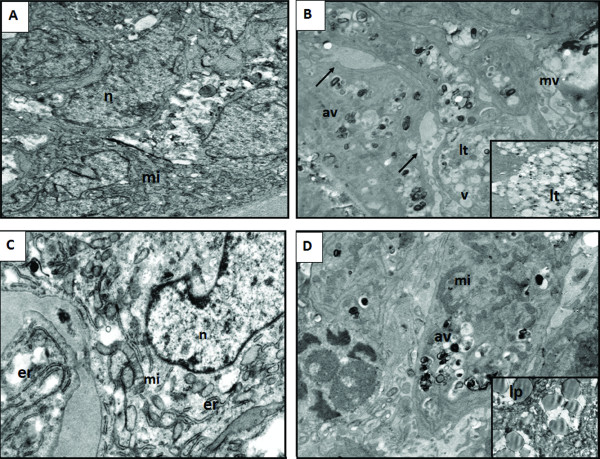
**Transmission electron microscopic views of T98G GBM spheroids at the 24^th ^hr**. **(A) **The control group (original magnification, ×7500). **(B) **The IM group (original magnification, ×7500; original magnification of small micrograph ×7500). **(C) **The Nos group (original magnification, ×10,000).**(D) **The combination group (original magnification of ×7500; original magnification of small micrograph ×10,000). **n**, nucleus; **mi**, mitochondria; **lt**, lytic cytoplasm; **av**, autophagic vacuole, **v**, vacuole; **(→): **disruptions of cell junctions; **mv**, microvillus; **lp**, lipid droplets; **er**, endoplasmic reticulum.

The control group exhibited normal morphology, characterized by fine-textured nuclear chromatin, intact nuclear membrane, tubular structured mitochondria, intact cytoplasmic membrane and many microvilli which were in contact with other cells under TEM (Figure 9A). Within the IM group, cells lost their microvilli and cell-to-cell connections, thus the integrity of spheroids was disrupted. This treatment group also exhibited severe mitochondrial damage, foamy-vacuolated cytoplasm, many presumably autophagic vacuoles and lytic changes. Apoptotic appearence was frequently seen in this group (Figure 9B). Nos led to severe mitochondria damage and expansion at endoplasmic reticulum. No autophagic vacuoles were observed. Apototic appearence was oftenly observed in this group (Figure 9C). The combination group exhibited presumably autophagic vacuoles which were lower than the IM group, mild mitochondria damage but healthy mitochondria was frequently observed and interestingly many lipid droplets in several sizes. The apoptotic appearence was rarely observed (Figure 9D).

## Discussion

In the present study, we used the combination of Nos and IM in order to potentiate cytotoxicity in monolayer cultured and spheroid cultures of human T98G GBM cells. The combination group showed antagonist effect leading to high cell proliferation index and low apoptotic index. IM was the most effective drug to inhibit cell proliferation and the latter was Nos.

Previous reports showed the inhibitory effect of Nos on GBM in vivo and in vitro [21,22]. One of them which was reported by Newcomb et al. demonstrated that Nos with IC_50 _value as 97 μmol/l inhibited T98G cell proliferation index in 24 hrs, induced M-phase arrest and led to a maximal cell death within 48 hrs [22]. In the present study, the maximal cell death was determined at the 24^th ^hr by using Nos with the IC_50 _of 10 μM. In addition, Nos induced S-phase arrest at the 24^th ^hr and G0+G1 arrest at the 72^nd ^hr (p < 0.05). Nos decreased EGFR, PDGFR-α levels and MRP-1 levels. According our pubmed search, the inhibitory effect of Nos on tyrosine kinase receptors (TKR) and MRP-1 hasn't reported yet.

The combination group induced highest decrease in cell proliferation and apoptotic indexes, caspase-3 levels, MRP-1 and PDGFR-α levels. The decrease in p170 levels were lower than IM but higher that NOS. The highest increase in EGFR, MK, bcl-2 and cAMP levels were determined in the combination group. The G0+G1 cell cycle arrest was the lowest in the combination group at the end of the 72^nd ^hr. The apoptotic appearence was rarely observed in morphological and ultrastructural evaluation. In addition, autophagic vacuoles were lower than the IM group. The underlying mechanism of antagonism seemed to occur because the combination group was unable to decrease anti-apoptotic proteins as EGFR, MK, bcl-2 and in addition it decreased G0+G1 cell cycle arrest and cytotoxic autophagy of IM. Consequently, the apoptotic index was decreased.

Phase I-II studies showed that IM is not active in GBM as they expected due to due to the fact that (1) a targeted agent was used in an unselected population which had receptor tyrosine kinase mutations and/or (2) a targeted agent was blocked by drug efflux proteins as commonly pronounced P-gp and MRP [27-29]. Previous studies showed that the inhibition of drug-efflux transporters enhanced cytotoxicity of several antineoplastic agents as a result of penetration improvement and drug distribution increase to the nervous system [29]. The highest decrease by the combination group was determined in MRP-1 levels. The combination group wasn't effective in the inhibition of P-gp levels, however Spiegl-Kreinecker et al. found that a considerable expression of P-gp was relatively rare in glioma cells, in contrast to MRP-1 which was constitutively overexpressed in cells derived from astrocytomas as well as GBMs as T98G and SW1088 cells [29]. In the present study, the inhibition of MRP-1 and P-gp was not enough to potentiate the cytotoxicity Mukai et al. also showed that IM is not a substrate for MRP-1 and IM didn't have ability to inhibit MRP-1 in leukemia cells. In addition, they proposed that IM would not enhance cytotoxicity in MRP-1 overexpressed cells when used in combination [30]. IM took second place to decrease MRP-1 levels and in the light of our results, we can propose that IM would enhance cytotoxicity in MRP-1 overexpressed cells.

Apoptotic appearence was observed rarely both in the morphologic and ultrastructural evaluation of the combination group. The autophagic vacuoles were frequently observed in the IM group. Autophagic vacuoles can be presumed as the first step of cell death because of high apoptotic index and the lowest bcl-2 levels. As Maiuri et al. used Bcl-2 inhibitor ABT-737 and they found that Bcl-2 inhibition led to free an autophagy inducer named beclin-1 [31]. In addition, Zhivotovsky and Orrenius suggested the cross-talk between cell death modalities that different signals can cause a shift from autophagy to apoptosis or apoptosis to autophagy or mixture of these two cell-death modes[32]. In our previous study which the combination of IM and chloripramine was tested in rat-derived glioma-C6 glioma cells, we considered that autophagy in the IM group may promote apoptosis [33]. No autophagic vacuoles were observed in the Nos group. In the combination group, the autophagic vacuoles were rarely seen. The combination group had the lowest apoptotic index and highest bcl-2 levels. We can speculate that the combination group couldn't inhibit bcl-2 levels and free autophagy inducer. Eventually, the autophagy couldn't occur and undergo apoptosis.

Broad success of MK inhibitors in preclinical studies make them highlighting targets in treating cancer [34]. IM induced the highest decrease and Nos led to mild decrease in MK levels, however the combination group increased MK levels at the end of the 72^nd ^hr. The antagonist effect of the combination group was related to high MK levels. In addition, Hu et al. reported that the MK gene may take part in multi drug resistance [35]. They showed that a powerful drug efflux ability correlated with high MK gene expression in lymphoblastic leukemia cells. In the present study In the present study, the combination group had the highest MK level but the lowest MRP-1 levels and lower P-gp levels. Thus, we can conclude that drug efflux ability was not correlated with MK levels in this experiment.

We also want to discuss the relationship between autophagy and MK. In the IM group we concluded above that autophagy was part of IM-induced cytotoxicity. Lowest MK levels were detected in the IM group, however the highest MK level were detected in the combination group. Consequently, we can speculate that MK can be modulator for cell death or cell survival. T98G control cells expresses MK highly and they are anchorage independent. We can also propose that T98G GBM cells escape from an anchorage dependent cell death anoikis because of the high MK activity. The underlying mechanism of this relationship was under investigation.

## Conclusions

The combination of Nos with IM showed antagonist effect in T98G human GBM cells in vitro. This antagonist effect was correlated highly with MK levels. The effects of NOS on MRP-1, MK and TKR levels were firstly demonstrated in our report. In addition, we proposed that MK is one of the modulator in the switch of autophagy to cell death or survival/resistance.

## Competing interests

The authors declare that they have no competing interests.

## Authors' contributions

ME was responsible for the conception and design of the study; ME and EA performed flow cytometric analysis of cell cycle and apoptosis; ME and AB carried out electron microscopy and cell culture studies; NY, EE and ME performed western blotting and ELISA studies; ME performed the collection and assembly of data, the statistical analysis and interpretation of data; ME wrote the manuscript; ME, AB, AS and VA revised the manuscript. All authors read and approved the final manuscript.
